# Supplementary material for: Predicting visual function by interpreting a neuronal wiring diagram
Source: Nature. 2024 Oct 2;634(8032):113–23. doi: 10.1038/s41586-024-07953-5 (PMC11446822; doi:10.1038/s41586-024-07953-5)
Supplement: Supplementary file 6 — CRF and ERF predictions for individual TmY4 and TmY9 cells. Analogous to Supplementary Data 3, but for TmY target types. Shown are the top four monosynaptic pathways, the strongest pathway passing through each of the top ten intermediary types (ranking from Extended Data Fig. 7), and the trisynaptic pathway Tm1–TmY–Dm3–TmY (see the section entitled Prediction of spatial normalization). [file 41586_2024_7953_MOESM6_ESM.zip › DataS4/TmY9qΓèÑ/720575940627304104.pdf]

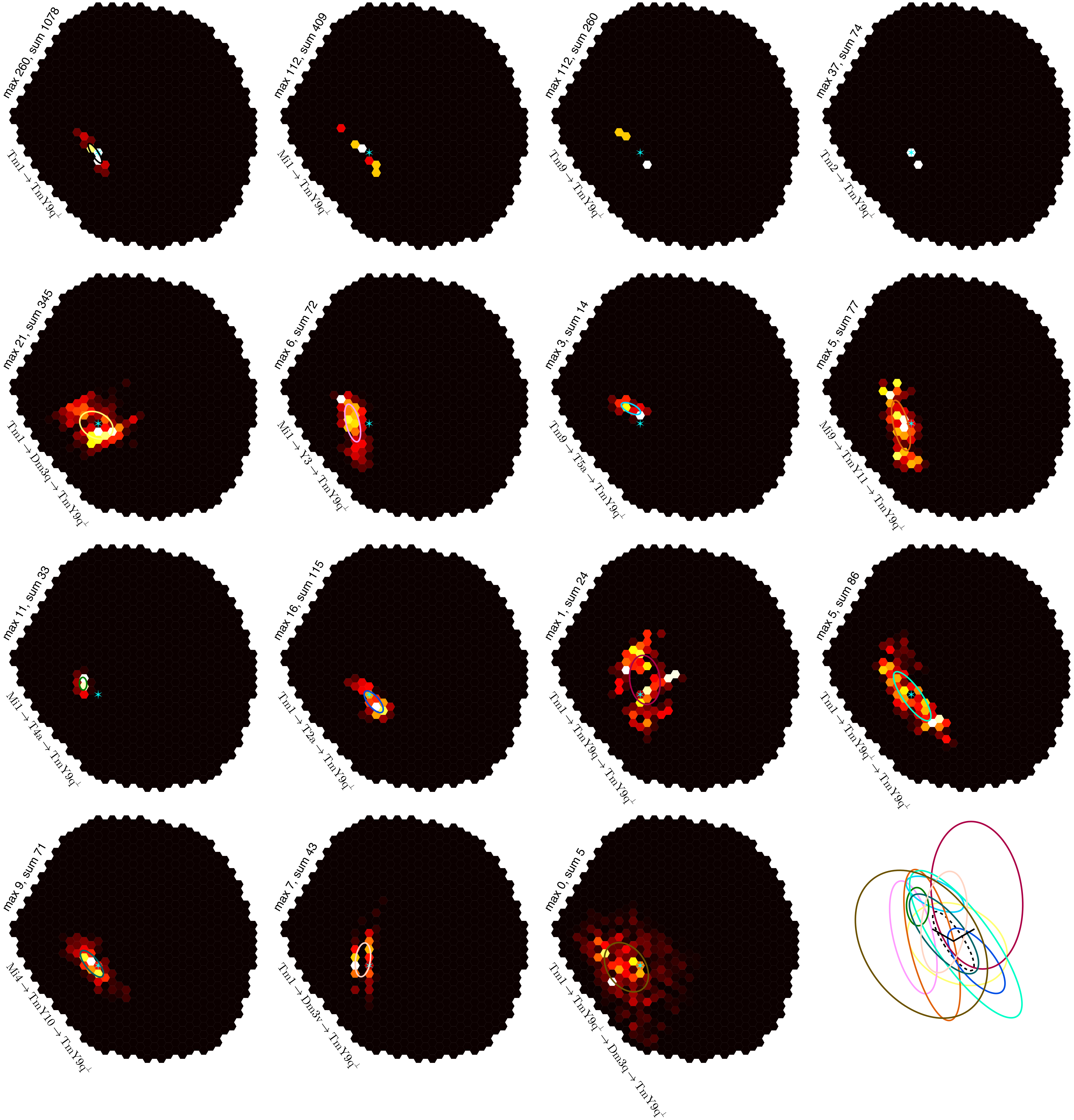

max 260, sum 1078
Tm1 → TmY9q⊥
max 112, sum 409
Mi1 → TmY9q⊥
max 112, sum 260
Tm9 → TmY9q⊥
max 37, sum 74
Tm2 → TmY9q⊥
max 21, sum 345
Tm1 → Dm3q → TmY9q⊥
max 6, sum 72
Mi1 → Y3 → TmY9q⊥
max 3, sum 14
Tm9 → T5a → TmY9q⊥
max 5, sum 77
Mi9 → TmY11 → TmY9q⊥
max 11, sum 33
Mi1 → T4a → TmY9q⊥
max 16, sum 115
Tm1 → T2a → TmY9q⊥
max 1, sum 24
Tm1 → TmY9q → TmY9q⊥
max 5, sum 86
Tm1 → TmY9q⊥ → TmY9q⊥
max 9, sum 71
Mi4 → TmY10 → TmY9q⊥
max 7, sum 43
Tm1 → Dm3v → TmY9q⊥
max 0, sum 5
Tm1 → TmY9q⊥ → Dm3q → TmY9q⊥
